# A Rare Case of Pulmonary Embolism, Deep Vein Thrombosis, Bilateral Avascular Necrosis of the Femoral Head, and Miscarriage following COVID-19 in a Patient with Multiple Genetic Coagulation Factor Deficiency—A Case Report

**DOI:** 10.3390/life13122240

**Published:** 2023-11-22

**Authors:** Nevena Georgieva Ivanova

**Affiliations:** 1 Department of Urology and General Medicine, Faculty of Medicine, Medical University of Plovdiv, 4000 Plovdiv, Bulgaria; nevenai@yahoo.com; Tel.: +35-98-8913-0416; 2 St. Karidad MHAT, Karidad Medical Health Center, Cardiology, 4004 Plovdiv, Bulgaria

**Keywords:** COVID-19, thrombotic complications, avascular necrosis, genetic defects of coagulation, bleeding

## Abstract

The coronavirus disease (COVID-19) is caused by the severe acute respiratory syndrome coronavirus 2 (SARS-CoV-2). The most common symptoms of COVID-19 are respiratory symptoms, but some patients develop severe thrombotic complications. Studies have looked into the association between the disease severity in COVID-19 patients and polymorphisms in the genes encoding prothrombotic and cardiovascular risk factors. The presented rare case describes inflammatory and acute thrombotic complications with musculoskeletal involvement in a patient with combined coagulation genetic defects. A 37-year-old woman was hospitalized with a respiratory infection of coronavirus etiology complicated by pneumonia and pulmonary embolism and confirmed using computed tomography and elevated D-dimer. Sixteen days after discharge, she developed deep vein thrombosis after discontinuation of antiplatelet and anticoagulant therapy due to bleeding. Four months after infection, we found bilateral avascular necrosis of the femoral head. The patient had a miscarriage with considerable blood loss and was given genetic testing, which confirmed the presence of a combined defect with a risk of both thrombosis and bleeding—heterozygous for the Leiden G1691A mutation, homozygous for the 677C>T mutation (MTHFR), heterozygous for the Val34Leu (factor XIII) mutation, and 4G/5G polymorphism in the promoter of the plasminogen activator inhibitor 1 (PAI-1) genes. The described rare clinical case poses a serious challenge regarding the anticoagulant and antiplatelet therapy, especially in the presence of thrombotic complications in COVID-19 and the underlying genetic defect associated with a risk of bleeding, including life-threatening intracranial bleeding. More research is needed to better understand the major medical concern about antithrombotic treatment in COVID-19 patients with bleeding risk in the context of genetic coagulation disorders. The case raises the vigilance of clinicians to search for a genetic predisposition to the development of severe thrombotic events in COVID-19 patients with no other known underlying diseases.

## 1. Introduction

The coronavirus disease (COVID-19) gained worldwide attention at the end of 2019, when a widespread outbreak of a severe respiratory infection with an unusually high mortality rate started in China. The etiological agent of this disease is a coronavirus, the severe acute respiratory syndrome coronavirus 2 (SARS-CoV-2). It contains ribonucleic acid and has the ability to mutate, thus altering the clinical course of the disease. Several variants of the virus have gained prominence so far: the Alpha (B.1.1.7), Beta (B.1.351), Gamma (P.1), Delta (B.1.617.2), and Omicron (B.1.1.529) variants [1]. The leading symptoms in all of them are upper and/or lower respiratory tract disorders [2] manifested in varying degrees of severity, and the febrile intoxication syndrome. As more information about the coronavirus disease has become known, it is now understood that some patients may experience severe thrombotic vascular complications that can worsen the prognosis and increase the disease’s mortality rate [3]. Studies have also looked into the association between the disease severity in COVID-19 patients and polymorphisms in the genes encoding prothrombotic and cardiovascular risk factors [4]. Musculoskeletal involvement can be another potential complication, which was less of a scientific focus at the start of the pandemic because it is not immediately life-threatening. In the post-COVID era, however, it is crucial to focus on this patient population as well, because they have decreased mobility and increased morbidity [5]. The pathophysiological mechanisms underlying these vascular complications have been suggested to be linked to vascular endothelial damage with subsequent endothelial dysfunction, excessive inflammation, intravascular coagulation, platelet activation and aggregation [6,7], corticosteroid treatment [8], and genetic predisposition [4].

I present here a patient with a rare combination of coagulation system-related genetic defects, which were found in the patient after the miscarriage she had. The patient subsequently developed several serious COVID-19 complications, including pneumonia, pulmonary embolism, deep vein thrombosis, and bilateral avascular necrosis of the femoral head.

## 2. Case Presentation

**Figure 1:**
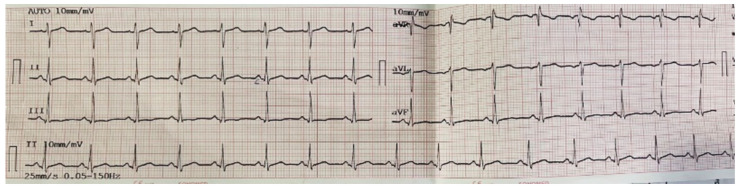

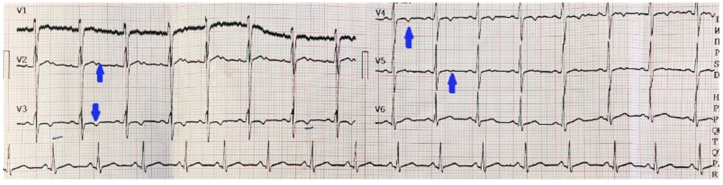
Electrocardiogram showing sinus tachycardia, right axis deviation, and negative T waves in V2–V5 precordial leads in pulmonary embolism.

A 37-year-old woman, with no known comorbidities, was admitted to the hospital with a 10-day history of fatigue, a 38 °C fever, a dry cough, and heavy chest pain. These symptoms had been treated unsuccessfully at home with ibuprofen 400 mg TID, paracetamol 500 mg TID, and vitamin C 500 mg QID as prescribed by a general practitioner. Three days before hospitalization, the patient began to secrete purulent sputum, experienced shortness of breath, chest pain, and severe adynamia with the slightest physical effort and had a resting heart rate of up to 120 beats per minute. The physical examination revealed that the patient was in poor general condition, had weakened vesicular breathing on the right base, and had tachycardic heart activity of up to 100 beats per minute. Laboratory tests showed abnormalities consistent with inflammation—leukocytosis (Leu 17.5 × 10^9^/L, normal range, 3.5–10.5 × 10^9^/L), increased C-reactive protein (CRP 68 mg/L, normal range, <5 mg/L), accelerated erythrocyte sedimentation rate (ESR 110 mm/h, normal range, 1–15 mm/h), elevated levels of D-dimer (D-dimer 2.37, >0.5/+/positive), and decreased oxygen saturation (OS 90%, normal range, 95–100%). In accordance with the viral infection, a reduced level of lymphocytes was observed at 0.80 × 10^9^/L and 17%, respectively (normal ranges 1.10–3.80 × 10^9^/L; 25–40% respectively). No abnormalities in the levels of thrombocytes were detected at 299 × 10^9^/L (normal ranges 150–450 × 10^9^/L). A PCR test was run in light of the patient’s clinical presentation, and the results showed that she was SARS-CoV-2 positive. Electrocardiogram (ECG) showed sinus tachycardia, right axis deviation (as a result of right-sided heart strain), and negative T waves in precordial leads V2–V5 (Figure 1).

**Figure 2:**
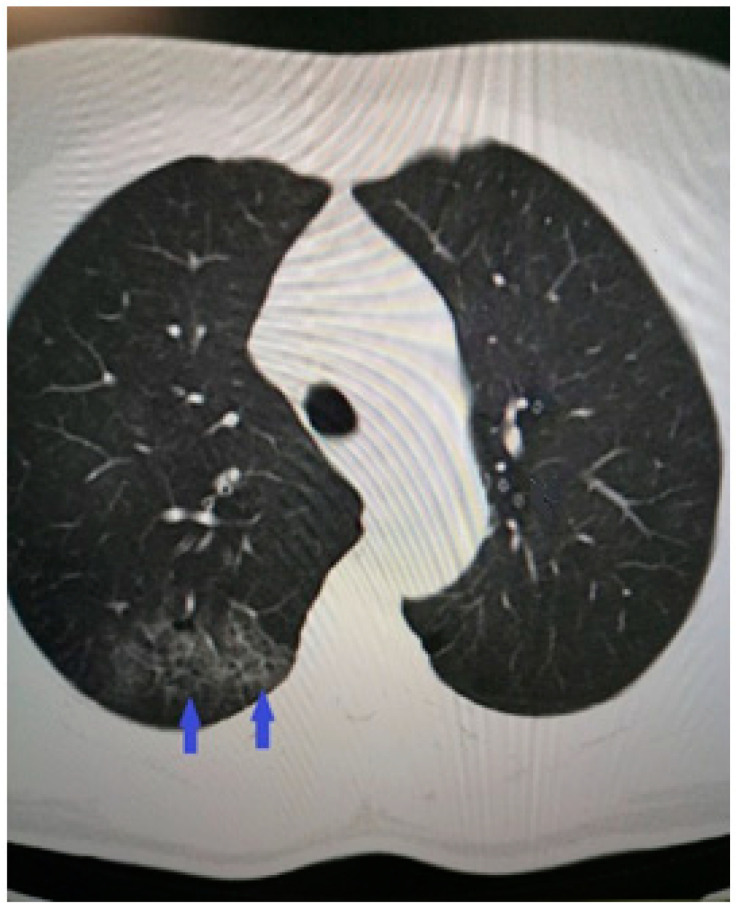
Chest CT showing peripheral right-sided consolidations and parenchymal ground-glass opacities consistent with COVID-19 pneumonia.

The diagnostic process continued with imaging studies. The computed tomography revealed right-sided pneumonia with COVID-19-typical ground-glass opacities and interlobular septal thickening (Figure 2).

**Figure 3:**
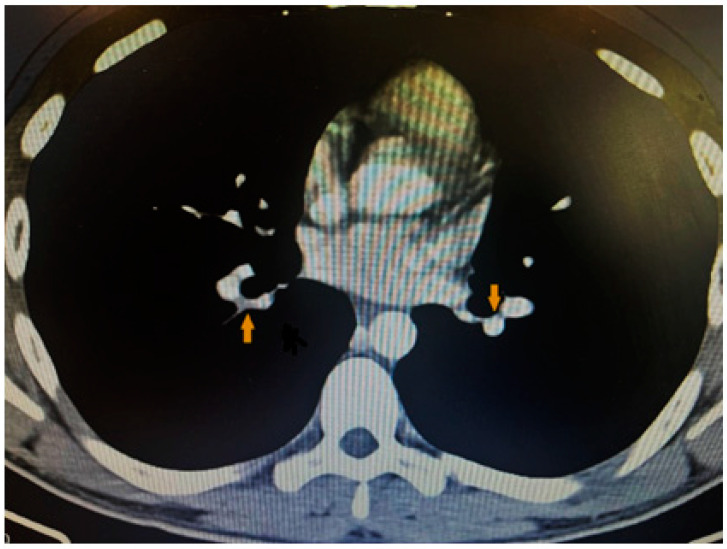
CT showing filling defects within the pulmonary vasculature with acute pulmonary emboli.

Considering the patient’s complaints of shortness of breath, the ECG changes, and the elevated D-dimer levels, as well as the suspicion of pulmonary embolism, we performed contrast computed tomography, which revealed inhomogeneity and a defect in the filling of small distal branches of the pulmonary artery—more to the left—which confirmed the diagnosis (Figure 3).

**Figure 4:**
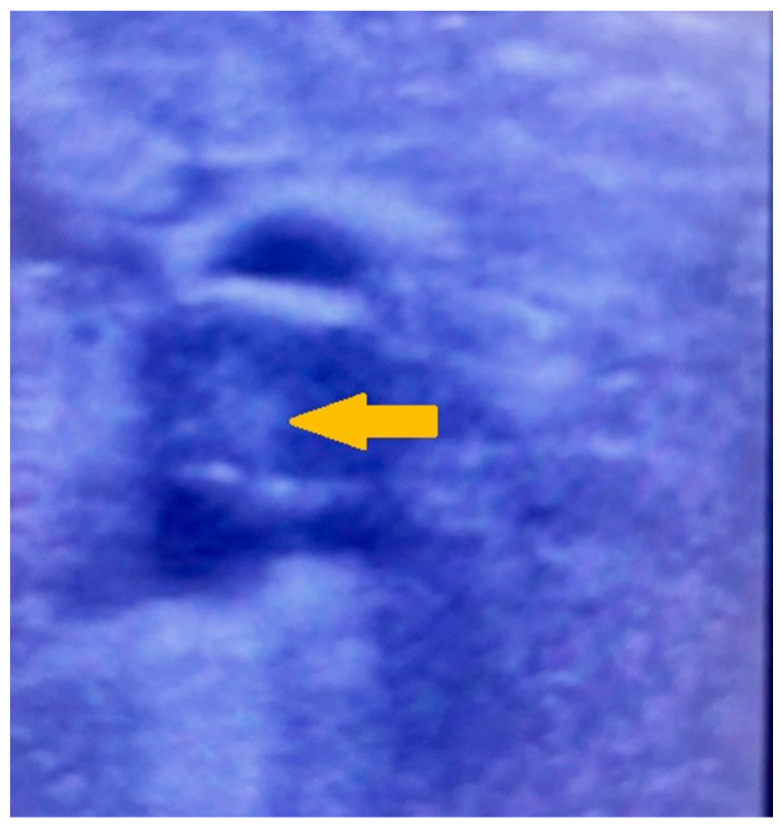
Ultrasonography showing thrombosis of the left femoral vein.

Treatment was started with oxygen supplied by a face mask at a flow rate of 5 L/h, antibiotics (meropenem, 1 g TID, and amikacin, 500 mg TID), corticosteroids (dexamethasone, a total dose of 168 mg for the time of hospital stay, equivalent to 1120 mg of prednisolone, and methylprednisolone at a total dose of 250 mg, equivalent to 312 mg of prednisolone), heparin infused at doses adjusted according to the levels of activated partial prothrombin time (APTT) for 2 days, followed by administration of low molecular weight heparin, nadroparin calcium (0.6 mL 2 times daily subcutaneously). The patient was discharged 10 days after being admitted with an improved general health condition and instructions to continue treatment at home with dexamethasone at a total dose of 78 mg (equivalent to 520 mg prednisolone), a proton pump inhibitor (pantoprazole 40 mg daily for 30 days), a non-vitamin K oral anticoagulant (dabigatran 150 mg twice daily), antiplatelet agents (clopidogrel 75 mg daily for 30 days, dipyridamole 25 mg 3 times daily 50 mg for 30 days), and antibiotic (azithromycin 500 mg daily for 10 days per os). After strictly following the prescribed course of treatment at home for 10 days, the patient stopped taking anticoagulants and antiplatelets due to heavy menstrual and epistaxis bleeding without seeking medical advice or having coagulation tests done. Six days later, the patient began to feel heaviness and discomfort in the lower left abdomen, which persisted for ten days with varying degrees of intensity. Within the following 24 h, she began to experience pain in her left leg during both active and passive movements, and the leg’s circumference grew. After consultation with her general practitioner, the patient was referred to and admitted to a vascular surgery unit. The physical examination revealed severe subfascial compression, pain on palpation in the left inguinal area, and preserved arterial pulsations in addition to massive swelling of the entire left leg with a volume difference of 5 cm for the lower leg and 7 cm for the thigh. The Homan’s sign test (dorsiflexon sign test) used to test for deep vein thrombosis (DVT) was positive/+++/. The laboratory testing showed no deviations in the level of thrombocytes 310 × 10^9^/L (normal range 150–450 × 10^9^/L). The initial diagnosis of deep vein thrombosis was confirmed by ultrasonography, which revealed thrombosis of the left femoral vein (Figure 4).

**Figure 5:**
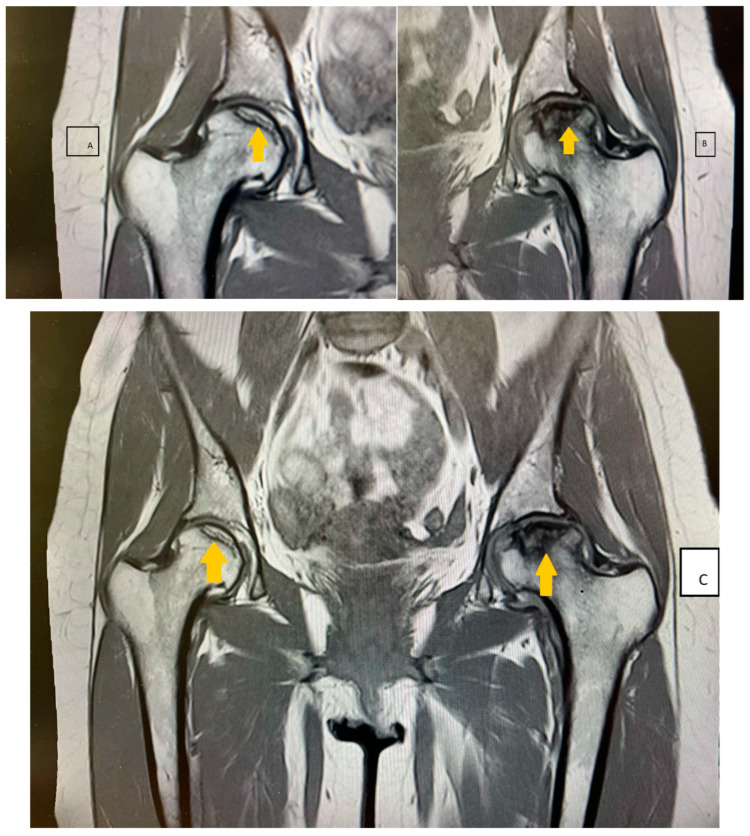
(**A**) The imaging study revealing focus of altered structure in the right hip in the pattern of initial avascular changes. (**B**)The femoral head of the left joint has deformed convexity and structure, with pronounced medullary edema of the neck and metaphysis, and an area of avascular necrosis measuring 2.24 cm^2^. (**C**) The femoral head was ultimately determined to have bilateral avascular necrosis.

The patient was treated with nadroparin calcium (0.6 mL 2 times a day subcutaneously for 12 days) followed by oral vitamin K antagonist acenocumarol (4 mg) in doses adjusted according to the PT and INR values, metamizole sodium (500 mg) or pitofenone hydrochloride (5 mg) or fenpiverinium bromide (1 tablet of 0.1 mg 3 times a day), and bioflavonoid vasoprotector diosmin (1 tablet of 1000 mg a day). Acenocoumarol was received for 2 months on a regimen and then discontinued again because of massive menstrual bleeding. Four months after the viral infection, the patient started to experience increased pain in her hands, knees, hips, and inguinal region. She also experienced morning stiffness that lasted for about an hour. The non-steroidal anti-inflammatory drugs the patient was taking had no effect. The motor deficit she had gradually progressed to the point of her being unable to move without crutches. A rheumatologist examined the patient and ordered an antinuclear antibody (ANA) test to rule out a systemic disorder. The ANA panel included tests for perinuclear anti-neutrophil cytoplasmic antibodies (p-ANCA), antineutrophil cytoplasmic antibodies (c-ANCA), anti-cardiolipin antibodies IgG, IgM (ACL-IgG, IgM), anti-phospholipid antibodies IgG, IgM (APL-IgG, IgM), anti-beta-2-glycoprotein I IgG, IgM, immunoglobulins IgG, IgA, IgM, and IgE, and complement components C3 and C4. These tests all came back within reference ranges, ruling out rheumatological disease, and the patient was referred to an orthopedist, who recommended a nuclear magnetic resonance examination. The imaging study found a focus of altered structure in the right hip in the pattern of initial avascular changes (Figure 5A). The femoral head of the left joint had deformed convexity and structure, with pronounced medullary edema of the neck and metaphysis, and an area of avascular necrosis measuring 2.24 cm^2^ (Figure 5B). The femoral head was ultimately determined to have bilateral avascular necrosis (Figure 5C).

The medication prescribed for the patient included a vasodilator (pentoxifylline 400 mg daily), vitamin D 10 drops daily, an antiplatelet agent (acetylsalicylic acid 100 mg daily), and a nonsteroidal anti-inflammatory drug (meloxicam 15 mg daily after meals).

**Figure 6:**
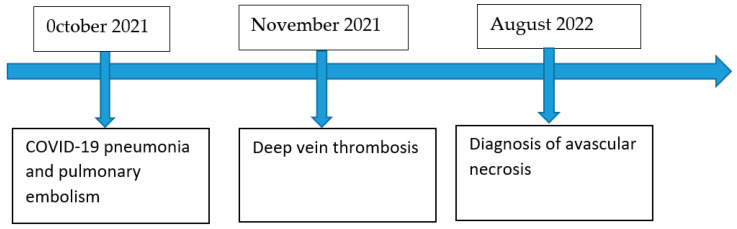
Timeline demonstrating the sequence of development of thrombotic complications.

Over a period of several months, the patient experienced various thrombotic events, including pulmonary embolism, deep vein thrombosis, and avascular necrosis with presumed similar pathogenesis (Figure 6).

After being diagnosed with avascular necrosis, the patient was referred to surgical treatment: replacement of the left hip joint. Two months after the diagnosis was made, the patient had a spontaneous miscarriage complicated by profuse bleeding.

Because the coagulogram we performed on this occasion showed deviations indicating an increased risk of bleeding—reduced prothrombin time (PT 64.8%, normal range 70–120%), high values of the international normalized ratio (INR 1.41, normal range 0.80–1.20), activated partial prothrombin time (APTT, 37.7 s, normal range 22.0–32.0 s), and fibrinogen (Fg 6.41 g/L, normal range 1.8–4.5 g/L)—the patient was referred to have genetic analysis at the suggestion of an obstetrician–gynecologist searching for a coagulation system-related underlying defect. Genomic deoxyribonucleic acid (DNA) was isolated from peripheral blood white cells using the “reverse” hybridization method. The patient was identified as heterozygous for the Leiden G1691A mutation, homozygous for the 677C>T mutation (MTHFR), heterozygous for the Val34Leu (factor XIII) mutation, and additionally typed for the 4G/5G polymorphism in the promoter of the plasminogen activator inhibitor 1 (PAI-1) genes. These studies demonstrated the presence of combined genetic defects that lead to an increased risk of both thrombosis and bleeding, which raises a question about the relevant therapeutic behavior in the future.

## 3. Discussion

SARS-CoV-2 caused the global COVID-19 pandemic in 2020, which put a tremendous strain on many healthcare systems around the world, increasing mortality rates and incurring huge economic losses. While COVID-19 is known to progress slowly in some people, with upper respiratory tract complaints being the major symptoms, in others, bilateral pneumonia or acute respiratory distress syndrome (ARDS) may develop. The patient I presented here experienced a number of inflammatory and acute thrombotic complications during the illness, including pneumonia and pulmonary embolism that required non-invasive ventilation therapy. Other vascular complications-related events, such as bleeding, deep vein thrombosis, bilateral avascular necrosis of the femoral head, and miscarriage, occurred in the months that followed. The genetic tests we ordered in connection with the miscarriage showed that the patient had a combined coagulation abnormality that put her at risk of thrombosis and bleeding.

Severe inflammation in acute infections is thought to be associated with a higher risk of thromboembolism [9,10], although the exact mechanism by which these events occur in COVID-19 is not completely understood. It is hypothesized that the acute infections with this condition induce an overproduction of cytokines (such as tumor necrosis factor (TNF), IL-1, IL-6, and IL-8) referred to as a cytokine storm, which results in enhanced intravascular coagulation, which, along with the altered immune response, leads to acute thrombotic consequences [11]. It is quite possible that vascular endothelial damage occurs, impairing the endothelial function as a result of the virus’s toxic effect on the cell induced by its entry into the cell via the angiotensin-converting enzyme (ACE) receptor [7]. In this way, the anticoagulant function is lost, which, together with other procoagulant factors such as the von Willebrand factor, leads to a hypercoagulable state. There is evidence that patients with severe COVID-19 have enhanced platelet aggregation, which increases the risk of thrombosis [12]. In an interesting study of 118 adult severe but non-critically ill COVID-19 patients, results have been presented of in vitro analysis of plasma thrombin generation potential and thrombophilia-related factors such as protein C and free protein S level. The data derived from the analysis of the thrombin generation curve showed a 33% longer lag time and 19% extended time to peak, which might be related to diminished thrombin generation potential in COVID-19. In addition, natural anticoagulants were found to be altered, with 17% lower protein C activity and 22% decreased free protein S levels, respectively [13], which could be addressed to the development of a hypercoagulable state in COVID-19 [13,14]. An observational study on 3334 patients hospitalized for COVID-19 found that 16% of the patients had a thrombotic complication during hospitalization, and 6.2% of whom developed pulmonary embolism [15].

Avascular necrosis (AVN) is defined as the cellular death of bone components, most commonly the epiphyses of the long bones (the femoral and humeral heads, as well as the femoral condyle), due to interruption of the blood supply, which leads to the collapse of the bone structure and impairment of the joint function. The pathophysiology of avascular necrosis is still unknown, although it is hypothesized that it has a multifactorial genesis with a common end result—interruption of the blood supply to the bone. It is most likely caused by the occurring vascular obstruction, ischemia, intraosseous hypertension, intravascular coagulation, or intraosseous extravascular compression that all restrict the blood supply to the bones resulting in necrotic alterations [16]. Corticosteroid-treated COVID-19 patients are more likely to experience adverse effects as a result of this medicine, but we should not exclude the possibility that the viral infection itself affects the musculoskeletal system [17]. Corticosteroids are used in this case to reduce inflammation, manage the cytokine storm, and prevent the organ failure the cytokine storm causes [18]. Although there are no reports in the literature of a precisely fixed dose threshold for corticosteroids known to induce avascular necrosis, some researchers suggest that a safe dose in such cases could be a 2000 mg cumulative dose of prednisolone or equivalent [19]. The total calculated dose of corticosteroids we used in the presented case was 1952 mg prednisolone or equivalent. Studies have demonstrated that the cumulative dose of steroid medication may be a better predictor for AVN than the daily dosage [20]. Given the evidence of the occurrence of coagulopathy and vascular thrombotic complications in COVID-19, it was assumed that the avascular necrosis in the patient was most likely triggered by SARS-CoV-2, and a genetic predisposition to these conditions was found later along with the additional effect of the administered corticosteroids, despite the fact that this medication did not reach the indicated cumulative dose of 2000 mg. The time of onset of avascular necrosis in our patient was about 4 months after the viral infection, while in 22 patients with COVID-19 in another study, the mean time of onset was 7.5 months. Similarly, the cumulative dose of corticosteroids taken by these patients was also less than 2000 mg, which led them to believe that the avascular necrosis was most likely caused by the virus-induced vasculitis with later femoral head necrosis [20]. It has also been found that it takes at least 6 to 12 months of corticosteroid treatment for symptoms of AVN to appear [17,20].

The likelihood of severe complications in COVID-19 varies among individuals; it is associated with the complex interaction between a number of underlying risk factors such as advanced age, male sex, and comorbidities including diabetes, obesity, hypertension, and heart disease [21,22], but it also depends on what genetic predisposition to the disease the specific individual has, which also contributes to the development of such complications [23]. Most of the genetic research in this regard focuses on studying the genes encoding angiotensin-converting enzymes 1 and 2 (the ACE1 and ACE2 genes) as the COVID virus is known to enter cells via these enzyme’s receptor. In contrast, there is very little research on the influence of individual prothrombotic genetic factors on the severity of the course of COVID-19 [4].

Factor V Leiden (FVL) G1691A is one of the most common inherited thrombophilias that raise the risk of abnormal thrombus formation and are associated with hypercoagulation. Factor V G1691A is a mutated protein that results from the replacement of guanine for adenine at position 1691, resulting in glutamate at amino acid residue 506 (R506Q) instead of arginine [24]. There is an allele associated with this type of defect that makes Factor V resistant to the proteolytic effect of protein C. As a result, the synthesis of the thrombin increases, resulting in a hypercoagulable condition. A study of 9508 thrombophilic patients with SARS-CoV-2 found that thrombotic complications occurred more frequently in patients who had FVL, and that this type of mutation is also thought to be correlated with disease severity [25]. Furthermore, a higher risk of thrombotic complications has been reported in adult SARS-CoV-2 carriers of the G1691A mutations in the FVL gene, despite administration of anticoagulants in therapeutic doses. This is the reason strict control should be applied over coagulation indicators and enhanced therapeutic regimens [26].

Methylenetetrahydrofolate reductase (MTHFR) is a gene that provides instructions for making the enzyme methylenetetrahydrofolate reductase, which breaks down homocysteine and folates. The presence of a genetic defect, the variant C677 T (c.677C>T), leads to a missense mutation in which alanine is replaced by valine (p. Ala222Val -rs1801133). In homozygous carriers of this variant, it leads to impaired homocysteine metabolism, which, in turn, is associated with vascular damage and prothrombotic conditions. A pathological correlation between homocysteine metabolism and COVID-19 has been addressed in several studies [27]. A very strong correlation has been found between the prevalence of the homozygous MTHFR-677 mutation and the morbidity and mortality of COVID-19; as such, this mutation might be employed as a biomarker to stratify the severity of COVID-19 infection and also to create a specific therapy strategy in these patients [28]. Carriers of this genetic defect may require a larger intake of folate and B vitamins to maintain normal homocysteine levels, which is why incorporating them as a preventative step in the therapeutic regimen may result in a lower mortality rate when infected with the coronavirus [29]. Other research also showed that there is an increased risk of thrombosis and complications [30].

Deletion/insertion polymorphism (4G or 5G) in the promoter of the plasminogen activator inhibitor type 1 (PAI-1) gene is suggested to be involved in regulating the synthesis of the inhibitor, 4G allele, being associated with the enhanced gene expression and plasma PAI-1 levels [31]. The 4G/5G polymorphism is associated with a higher risk of venous thrombosis [32,33] and femoral necrosis [34]. Moreover, other authors have looked into the influence of the genetic mutation plasminogen activator inhibitor (PAI)-1 4G/5G and coronavirus infection. Data from a study on 47 patients with COVID-19 indicate that this type of polymorphism may be a predictor of the severity of the course of the viral infection [35]. Other authors also found an association between the 4G/5G polymorphism and osteonecrosis arising from intravascular thrombosis and the formation of fibrin thrombi in the area of osteonecrosis [36]. In addition, a meta-analysis including 22 studies with 4306 cases and 3076 controls was published where the authors concluded that PAI-1 4G/5G polymorphism might be associated with recurrent pregnancy loss development in Caucasians [37].

Factor XIII is a proenzyme activated by thrombin in the coagulation cascade. It catalyzes the formation of the fibrin clot and has a crucial role in the stabilization of fibrin networks, thereby preventing premature fibrinolysis. A genetic defect encoding a valine (Val) to leucine (Leu) substitution at codon 34 (Val34Leu) was found in 25% of European Caucasians [38]. Factor XIII deficiency leads to a risk of bleeding and the development of a hemorrhagic diathesis. Moreover, the Leu34 allele has been pointed out as a risk factor for intracerebral hemorrhage [39]. In addition, data derived from a study of 102 patients with COVID-19 have shown an intense proportional decrease in Factor XIII plasma levels, which has been related to the level of respiratory support. Furthermore, these levels were extremely lower in patients who died within 30 days of hospitalization than in others who survived. The presumable mechanism of acquired Factor XIII deficiency is that it is consumed in the ongoing activation of coagulation even with the anticoagulant treatment received. It has been suggested that these alterations contribute to COVID-19-related bleeding [40]. The described patient was found to be heterozygous for the Val34Leu (Factor XIII) mutation, which further increases the risk of bleeding, especially when combined treatment with anticoagulant and antiplatelet agents is administered.

A number of studies have demonstrated that COVID-19 and thrombophilia are correlated, making screening for thrombophilia in SARS-CoV-2 patients beneficial to decreasing mortality [41,42,43].

## 4. Conclusions

The presented rare clinical case demonstrates several of the possible complications of SARS-CoV-2 infection, in a patient with subsequently proven combined genetic defects affecting the coagulation system, associated with and leading to simultaneous thrombosis and bleeding. These are, on the one hand, the inflammatory complications in which pneumonia develops, and, on the other, the thrombotic complications in which pulmonary thromboembolism and deep venous thrombosis may occur. I could also associate avascular necrosis with the combined effect of coronavirus and the genetic factors that impact coagulation and hence the trophism of the femoral head, but also with the corticosteroids the patients received, although a cumulative dose was not reached. The described clinical case presents a significant challenge in terms of the anticoagulant and antiplatelet therapies, particularly in the development of thrombotic complications and their prevention in COVID-19, because the patient also has an additional genetic defect associated with the risk of bleeding, including life-threatening intracranial bleeding. More research is needed to better understand the major medical concern about antithrombotic treatment in COVID-19 patients with bleeding risk in the context of genetic predisposition to both coagulation disorders. The case raises the vigilance of clinicians to search for a genetic predisposition to the development of severe thrombotic events in COVID-19 patients with no other known underlying diseases. In addition, practitioners may benefit from screening for thrombophilia in patients with SARS-CoV-2 in terms of choosing the most appropriate therapeutic strategy and thus influencing patients’ prognosis.

## Data Availability

The data presented in this study are available on request from the corresponding author. The data are not publicly available due to privacy/ethical reasons.
